# How Job Demands and Resources Relate to Experiences of Bullying and Negative Acts among University Employees

**DOI:** 10.3390/ijerph19148460

**Published:** 2022-07-11

**Authors:** Gunhild Bjaalid, Elena Menichelli, Dian Liu

**Affiliations:** 1 Department Health Technology, University of Stavanger, 4021 Stavanger, Norway; 2 Department Business Administration and Development, University of Stavanger, 4021 Stavanger, Norway; elena.menichelli@uis.no; 3 Department Media and Social Science, University of Stavanger, 4021 Stavanger, Norway; dian.liu@uis.no

**Keywords:** workplace bullying, higher education, negative acts questionnaire, JD-R model

## Abstract

This article addresses a gap in the work psychology literature regarding psychosocial working conditions and bullying among staff in academic organizations. We examine the influences, institutional demands, and resources attached to given academic positions, such as how the level of social support and cooperation influence the level of experienced negative acts at work and bullying in different work groups in an academic work environment. We also examine whether some professions or positions in an academic organization are more vulnerable due to organizational structure, perceived and experienced resources, and demands to bullying or experiencing more negative acts at work. A common division of different employees in the university sector is between administrative/technical staff and scientific personnel. Our hypothesis in this study is that there are significant differences among these two groups regarding levels of experienced social support and cooperation, as well as levels of experienced negative acts at work. We postulate that differences in job demands and resources lead to significantly different levels of self-reported bullying for the two main groups of personnel. We expect scientific personnel to be more exposed to negative acts at work and bullying due to differences in the demands and resources associated with these positions.

## 1. Introduction

This article addresses a gap in the work psychology literature regarding the psychosocial working conditions and bullying among personnel in higher education organizations in the Nordic context. Academic organizations and universities all over the world have a bad reputation for offering their employees a rough work environment, with high levels of competition among employees and prolonged and unsolved conflicts in combination with low levels of social support and cooperation among employees, leading to high levels of workplace bullying [1,2,3].

The relatively high incidence of bullying in the university sector has been attributed to specific social and work features of the academic profession [4]. Academic organizations have been described as high on role overload, low on job feedback, low on participation in important organizational decisions, and low on recognition and rewards practices [5]. A longitudinal study from a medical university in Sweden [6] indicated that a poor social and innovative work climate, unfair and disempowering leadership, incompatible work roles, and a lack of support from superiors and colleagues were the factors most strongly related to future bullying in a group of scientific and administrative personnel. Many universities also offer scientific personnel low job safety in the sense that permanent positions are rare and hard to obtain. It has been claimed that typical academic organizations offer their members a *survival of the fittest* type of work environment [7], and only the toughest and most resilient manage to stay, develop, and shine in an academic work setting.

A university can almost be compared to a micro-society in that it contains a wide range of different professions and positions with a range of different backgrounds and educations. This paper studies the employees charged with the day-to-day operations of a university—from human resource professionals, financial operators, and IT professionals to the more traditional scientific positions such as professors.

Conflicts are a natural part of everyday life in organizations. Interpersonal conflicts in the work environment are a common problem in most sectors, and conflicts are also one of the main reasons for reduced psychosocial work conditions. In a study on living conditions in Norway, 30% of the respondents reported being in a conflict with their manager or department head, and 20% reported being in a conflict with a colleague [8]. Conflict resolution is reported to take approximately 20% of a manager’s time [9].

The phenomenon of workplace bullying refers to a prolonged and repeated negative behaviour at work directed against employees who are unable to defend themselves [10], leading to numerous negative outcomes for the affected workers and for the organizations [11,12]. A single negative act is typically not considered bullying, but a pattern of negative acts over a prolonged period can be considered bullying. Bullying is seldom explained by one factor alone but is rather a multicausal phenomenon [13].

Self-reported workplace bullying in Norway is normally reported to be around 5%, depending on the sector [8]. In a national survey including 42,778 employees in the university sector in Norway [14], the average self-reported bullying score among the respondents was 13%. These numbers indicate that university employees have a considerably higher risk for experiencing bullying and negative acts at work in Norway than employees in other work sectors.

### 1.1. The Nordic Academic Work Sector

Scandinavian leadership has historically been characterized by three central features, namely a high level of employee involvement in the decision-making process, a short distance between managers and employees, and “freedom with responsibility”, which means that the employee has ample space in which to take initiative and work independently [15]. This will most likely also affect the leadership style of the Nordic universities.

In the Nordic academic work sector, scientific personnel, especially professors, have had considerable amounts of autonomy [16]. This autonomy has been challenged during recent decades by the introduction of management standards in European university systems [17]. Additionally, the global forces in academia also play a role in affecting academic autonomy and decentralization [18]. A move towards becoming a more business-like organization, where everything is weighed and measured, has challenged the everyday management of Nordic universities.

### 1.2. The Job Demand Resources Theory

According to the Job Demand Resources theory [19], also called the JD-R model, job demands are initiators of a health impairment process, and job resources are initiators of a motivational process. The model also specifies how certain demands and resources interact to predict organizational outcomes such as organizational commitment and job engagement.

One central assumption of the JD-R model is that every occupation may have its own specific work characteristics associated with both job engagement and job exhaustion. It is, according to the JD-R model, possible to model these characteristics into two broad categories, job demands and job resources [19].

The JD-R model defines job demands as physical, psychological, social, or organizational aspects of the job that require sustained physical and/or psychological (cognitive and emotional) effort and are therefore associated with physiological and/or psychological cost. Examples can be a poor psychosocial work environment, high workload, or conflicting roles. Job resources however are defined as the physical, psychological, social, or organizational aspects of the job that function in achieving work goals, reducing job demands associated with high physiological and psychological costs, and stimulating personal growth and development.

In academic organizations, resources can be related to external or internal research funding possibilities, administrative support, and access to Ph.D. candidates. Resources may also be found in interpersonal and social relations in the work setting such as support from leaders or co-workers and a good psychosocial work environment in general. The organization may also provide resources by offering high degrees of job clarity and good leadership competence and by allowing employees to participate in important decisions regarding their work situation.

Job demands in an academic organization can be both positive and negative. Positive demands are closely related to meeting difficult goals [20]. According to the JD-R model, having overly complex and difficult work tasks, such as high work pressure regarding publications in the most prestigious journals, teaching and student responsibilities, and the acquisition external fundraising, on top of high levels of conflict and low levels of social support, can result in experiencing a higher degree of demands than resources in an academic work setting [21,22].

According to the JD-R model, job demands will normally require the use of resources. If, as an example, a researcher works in a department with a high level of interpersonal conflicts and low social support, this will normally preclude the mobilization of job resources [21]. If employees are in a work situation with prolonged demands and lack of resources, a reduction of work motivation and withdrawal from work can, according to the JD-R model, be an important self-protection strategy that may prevent future frustration from not meeting work-related goals [23]. Exhaustion and cynicism or disengagement from work, seen as core dimensions in burnout, can be observed in virtually any occupational group [24].

The JD-R model assumes that high job demands such as a high workload and a high level of role conflict and low levels of resources such as task autonomy and social support increase bullying over time. An empirical study including 177 employees from various establishments in Belgium supported this hypothesis [25]. The results showed that job demands were positively related to targets’ reports on bullying one year later, and job resources were negatively related to targets’ reports of bullying.

In this article, we focus on how job resources and demands, such as high levels of reported social support (resources) and low levels of cooperation among employees (demands), affect the level of reported negative acts and bullying among two different groups of employees. We tested the relationship between job demands and job resources and how it affects the types and extent of negative acts in an academic organization. We measured job demands in terms of quantitative demands, lack of feedback and cooperation, and institutional stress, and we measured job resources by self-reported social support and cooperation, meaningful job tasks, and autonomy.

We distinguished between scientific and administrative personnel because the JD-R model focuses on the importance of awareness that every profession and work climate may have their own unique pattern of typical expected demands and available resources built into the organizational structure. Administrative and technical personnel in our sample include both employees working in different departments at the faculties, and employees located in centralized departments such as HR, the communication department, the economy department and the IT department. Administrative and technical personnel is therefore a term for all employees who do not have a scientific position at the university.

Another proposition of the JD-R model is that several different job resources can play a buffering role for several different job demands, making a simple model more complex. Not only will a work environment have its unique pattern of demands and resources built into the job design, but so will the different professions and occupations and every individual employee. Consistent with this view, local and thorough information is needed to design healthy and productive work environments. Therefore, it is also meaningful to explore what type of job demands and resources can act as a buffer or a risk for bullying in different work environments and for different types of positions.

This study also provides data to test an empirical model of organizational and structural factors leading to higher risks of bullying in an academic work setting.

We were interested in examining if any of the different positions at a university—due to the type of work tasks they perform, how the work processes are structured and organized, or the type of cooperation and dependability on other professions they have at work—are more at risk of experiencing higher degrees of negative acts or bullying due to the patterns of job demands and resources offered by their organization.

**H1.**  *Structural organizational resources and demands predict the level of workplace bullying among university personnel.*

**H2a.**  *Scientific staff as a group experience different effects of demands and resources on bullying than administrative staff.*

**H2b.**  *The effect of demands on bullying is stronger for the scientific staff than for the administrative staff.*

**H2c.**  *The effect of resources to prevent bullying is stronger in the administrative group than in the scientific group.*

## 2. Materials and Methods

### 2.1. Data Collection

The data used in this study were collected using a work environment questionnaire survey that was sent to all employees at a university on the west coast of Norway in the spring of 2019. The survey included a range of validated questionnaires on themes relevant to the above issues. In total, 1600 employees received the questionnaire, and the overall response rate was 84% for the total sample of employees.

To eliminate the respondents that were returning a large proportion of missing values, only the returned surveys in which more than 50% of the questions were answered were kept. This criterion led to 922 answered surveys. Of those, 450 were administrative/technical staff working in support positions, 311 were scientific personnel with both research and teaching duties at diverse career stages, and 161 covered other types of positions and were not specifically considered in this study.

#### 2.1.1. Measurements

In this study, job demand variables included quantitative demands, lack of feedback and cooperation, and institutional stress. Accordingly, *Quantitative demands* were measured using QPS-Nordic [26,27] with the following items: (1) Is your work unevenly distributed so it piles up? (2) Do you have to work overtime? (3) Do you have to work very fast? and (4) Do you have enough time for your work tasks? *Lack of feedback* was measured by a questionnaire from a Nordic research group [28] with the following items: (1) In my work, there are many opportunities to judge how well I am doing my work, (2) My boss(es) often tell me they appreciate me doing my work tasks, and (3) It is easy for me to see the positive effects of my work. *Lack of cooperation* was measured by the cooperation scale from QPS-Nordic and Census work environment questionnaire [26,27] by the following items: (1) Have you noticed disturbing conflicts between work colleagues? (2) When conflicts arise, are they handled well? (3) Do you find that the collaboration at your department/in your unit/on your management team works well? and (4) Do you find that your department/unit/management group is characterized by security and trust? *Institutional stress* was measured using Cooper’s Job Stress Questionnaire (CJSQ) [29] with 5 items assessing institutional stress [30]. The CJSQ combines different types of work-related stress and assesses stress using various elements rated on a six-point scale ranging from no stress to very much stress. Items used to measure institutional stress were: How much work-related stress have you experienced concerning the following? (1) The organization’s policy, (2) Lack of power and influence, (3) My values conflicting with those of the organization, (4) The leadership not understanding the challenges of my work, and (5) The organization using the wrong parameters to measure the quality of my work.

*Job resources* include autonomy, self-reported social support, and meaningful job tasks. *Autonomy* was measured using the autonomy scale from the Organization Assessment Survey [31] with the following items: (1) In my department, we work together to influence the standards that constitute good work, (2) In my department, we often have the opportunity to influence goals or actions, (3) All employees in my department are involved in important decisions that affect them, and (4) Employees have good opportunities to influence how work is carried out. The questions were measured on a five-point scale ranging from to a very small extent to a great extent. *Social support* was measured by a scale developed by Van der Heijden [32] with the following items: (1) Are your colleagues able to appreciate the value of your work and see the results of it? (2) Do your colleagues express their opinions about your work? and (3) Do your colleagues offer constructive advice? *Meaningful job tasks* were measured with the “positive challenges at work” scale from the QPS-Nordic instrument [26]. The items included the following questions: (1) Are your skills and knowledge useful in your work? (2) Is your work challenging in a positive way? and (3) Do you consider your work meaningful?

#### 2.1.2. The Dependent Variables

*Bullying*. Exposure to workplace bullying was measured using a 12-item trimmed version of the Negative Acts Questionnaire—Revised (NAQ-R) [33]. All items are formulated in behavioural terms, with no reference to the term bullying, and the items refer to both direct (e.g., verbal abuse) and indirect behaviour (e.g., withdrawal of information). A list of negative acts in the workplace was presented (see table below), and the answers were on a 5-point scale (No, Rarely, Now and then, Once a week, Several times a week).

*Self-reported bullying* was measured by the item “Have you been exposed to bullying at your workplace during the last six month?” [33].

### 2.2. Statistical Analyses

#### 2.2.1. The Relation between Pre-Bullying and Self-Reported Bullying

We first ran a principal component regression (PCR) on the pre-bullying variables to predict self-reported bullying, in order to test whether negative acts in the workplace give strong indications about actual bullying and whether previous validations of the NAQ-R, as a two-factor measurement of work-related and person-related bullying [33], are confirmed in this case study.

#### 2.2.2. The Interpretation of the NAQ-R and the Other Measurements

To understand whether the administrative and scientific staff have a different perception of negative acts, we first considered the number of employees in the two groups who reported having been exposed to some negative acts at the workplace (i.e., the ones who answered ≥2 on a 1–5 scale). We then tested whether the percentage of employees stating so was significantly different (this was achieved by calculating the comparative error, and the significance was tested by checking whether the percentage difference between the two groups was greater than the comparative error.)

The dimensionality underlying the NAQ-R questions was also investigated through a factor analysis, the extraction method being a principal component analysis with varimax rotation. This compresses the variability into new few factors that are non-correlated and are thus linear combinations of the original variables. The same was performed on the measurements for job demands and resources. The new factors could thus be used in modelling methods such as structural equation modelling (SEM) (explained below).

#### 2.2.3. Modelling the Effects of Resources and Demands on NAQ-R

SEM was used to uncover possible causal effects on negative acts, thus allowing the estimation of a theoretical network of relationships linking latent complex concepts (resources, demands, negative acts), each measured by means of observable indicators (factors from the factor analysis on the original variables). The chosen SEM methodology is component-based so as to maximize the amount of variance explained and is referred to as a Partial Least Squares Path Model (PLS-PM) [34]. In addition, PLS-PM is chosen because it serves an exploratory approach to new theory testing and refers to “soft modelling” (few distribution assumptions, i.e., few cases can suffice, which is useful when creating subgroups). PLS-PM also has the advantage of handling models where the latent concepts can be either/both reflective or/and formative. A PLS-PM is described by a measurement model, relating the manifest variables to their own latent variable and a structural model, relating endogenous latent variables to other latent variables. The latent variables in this model are generated by their own manifest variables in a formative way (i.e., the latent variable is a linear function of its manifest variables plus a residual term). In the formative model, the block of manifest variables can be multidimensional, as it is the case for demands, resources and negative acts in the workplace: the latent variables include independent factors from factor analysis, with the extraction method being the principal component analysis. The validation of the total model is given by the goodness of fit (GoF), meant as an index that is looking for a compromise between the performances of the measurement and the structural model [35].

**Figure 1:**
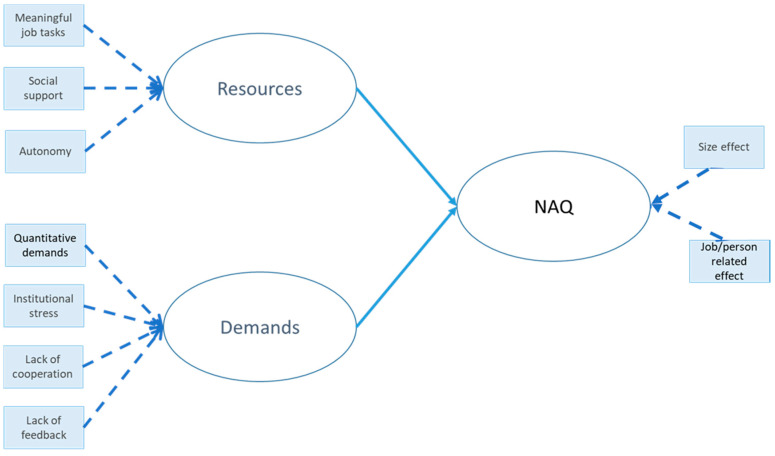
Model specification for the PLS-PM, which is described by a measurement model relating the manifest variables to their own latent variable and a structural model relating some endogenous latent variables to other latent variables.

Our model specification (Figure 1) aimed at testing the hypotheses described in the JD-R theory section. The measurement model related the factors obtained from factor analysis in the following way: (i) the manifest variables “meaningful job tasks”, “social support”, and “autonomy” were related to their own latent variable “Resources”; (ii) the manifest variables “quantitative demands”, “institutional stress”, “lack of cooperation”, and “lack of feedback” were related to “Demands”; and (iii) the two “negative act factors” were related to the latent concept “NAQ”. For the measurement model, the manifest variables for “Resources” and “Demands” were related to their own latent variable in a formative way. The latent concept “NAQ” was created in a formative way; thus, the block of manifest variables can be multidimensional (as they were constructed by principal component analysis). The assessment of formative measurement models involves the test for potential multicollinearity between items (variance inflation factors values below a threshold) and the analysis of weights, which provide information about how each item contributes to the construct measurement. The structural model related the endogenous latent variables “Resources” and “Demands” to the dependent latent variable “NAQ” through direct effects. This followed the JD-R model in the assumption that high job demands and low levels of resources increase bullying.

The model specification was applied to the whole survey sample as well as to the scientific staff and to the administrative staff separately. This was useful for validation purposes in terms of robustness of the results and for testing differences between the two groups of employees.

## 3. Results

### 3.1. The Relation between Pre-Bullying and Self-Reported Bullying

The PCR on the pre-bullying variables to predict self-reported bullying performed well (R^2^ = 0.42). Our results (Table 1) indicate that most of the pre-bullying variables had a significant effect on bullying (no multicollinearity was detected). The three most significant variables were: having slanders and rumours spread about you, being exposed to exaggerated teasing or malicious kidding, and being overlooked or excluded from social relationships. Because the first factor from PCR was strongly correlated with self-reported bullying (r = 0.62) and was significant in predicting it, we can state that negative acts in the workplace give strong indications about actual bullying. Our results thus support previous validations of the NAQ-R as a two-factor measurement of work-related and person-related bullying [33].

### 3.2. The Interpretation of the NAQ-R and the Other Measurements

Considering the number of employees who reported having been exposed to some negative acts at the workplace during the past 6 months, the results showed that scientific and administrative personnel experienced significantly different levels of negative acts and self-reported bullying (Table 2a). These values were also significantly different for five of the twelve NAQ-R questions (Table 2b), these five questions being mainly person-related: Hostility or silence in response to questions or attempts at conversation (NAQ-6), Having your opinions ignored (NAQ-7), Having gossip and rumours spread about you (NAQ-8), Having insulting or offensive remarks made about your person, attitudes, or private life (NAQ-9), and Being ignored or excluded from the social community at work (NAQ-11). The percentage difference between the groups indicated that the scientific staff had consistently higher bullying values than the administrative staff.

Table 2a shows that there was a significant difference in response between the two groups of employees for both self-reported bullying and observed bullying from other colleagues. Table 2b indicates the negative acts at the workplace that the scientific group had experienced at a significantly higher rate than the administrative group. The scientific staff showed consistently higher values than the administrative staff.

The dimensionality underlying the NAQ-R questions was investigated by factor analysis, which showed that the first factor accounted for as much variance as about six original variables. The scree plot (not shown) also suggested keeping the second factor because the slope of the curve was levelling off after it and because it was an important factor according to interpretation. These two factors explained a sufficient percentage of the total variability (59%) and were thus kept for further analysis. (The other four factors had an eigenvalue that was smaller than 1 and did not show relevant interpretation features, and thus, they were discarded.)

The eigenvectors (i.e., the coordinates of the variables in the new factor space) indicated that the first factor explained the size effect of the NAQ-R. The second factor was instead discriminating between undesirable actions at the workplace that were either at the personal level or were work-related (see Table 1), in general confirming the categorization by Einarsen et al. [33]. The extremes of this “bipolar” dimension were the person-related variables NAQ-10 (Being the subject of excessive teasing and sarcasm) and NAQ-12 (Practical jokes carried out by people you do not get along with), and the work-related variables NAQ-4 (Having been deprived of important responsibilities, being ordered to do work below your level of competence) and NAQ-1 (Someone withholding information that affects your job performance). In addition, each of the measurements was well explained by their first factor from factor analysis because Cronbach’s alpha was greater than 0.7, and there was sufficient explained variability (not shown). The new factors were used in an SEM model to fit our JD-R model assumption.

### 3.3. Modelling the Effects of Resources and Demands on Negative Acts

#### 3.3.1. General Model

**Figure 2:**
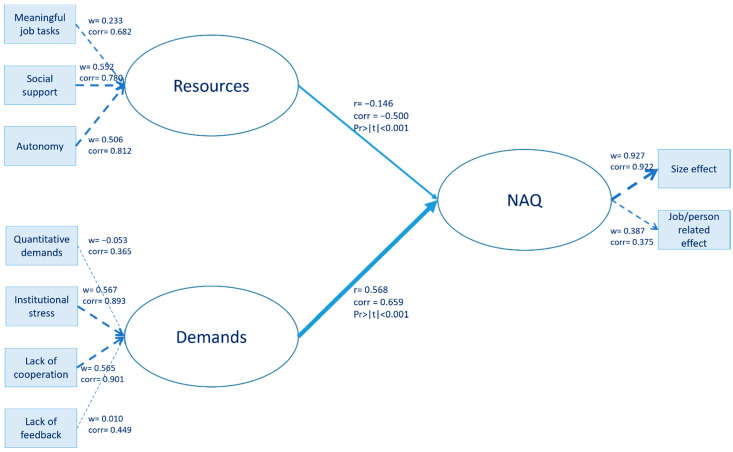
SEM results for the whole sample of employees. Structural organizational resources and demands significantly predicted the level of workplace bullying among university personnel.

When considering the SEM structural model for the whole sample of employees (Figure 2), both resources (with a negative sign) and demands (with a positive sign) had a highly significant effect on negative acts at the workplace. The greater the demands, the more negative acts at the workplace, and the more resources, the fewer negative acts at the workplace. The effect of demands played an especially big role, being about four times stronger than the effect of resources on bullying.

These results indicated that the latent variable for demand was mainly constituted by the observed variables for institutional stress and lack of cooperation in terms of both weight and correlation. Resources were mainly formed by social support and secondly by autonomy. Additional SEM models for the whole sample of employees, as well as for the administrative and scientific staff separately, were tested by discarding the size-effect factor for the NAQ-R and by focusing only on either work-related or person-related factors (obtained by factor analyses of the corresponding original NAQ-R variables). The results from these six different models, not shown here, were quite robust and did not highlight the different weights or constructs in the measurement models for resources and demands. They confirmed the patterns that we detected from the SEM models described in this paper.

The assumption of the JD-R model was thus confirmed, and high job demands, such as a high workload and lack of cooperation, and low levels of resources, such as low task autonomy and lack of social support, increased negative acts and bullying in the workplace. Therefore, these results support hypothesis H1: structural organizational resources and demands predict the level of workplace bullying among university personnel.

The quality of a PLS-PM depends on the GoF of both the measurement and structural models. For the measurement model, the test for potential multicollinearity between items was satisfied because the maximum variance inflation factor value was 1.833. The discriminant validity was also satisfied (squared correlations always smaller than mean communalities, not shown).

For the structural model, the average R^2^ (measuring the predictive performance) was satisfactory, being equal to 0.447 (Table 3a). The contribution to R^2^ was equal to 14% for resources and 86% for demands (Table 3b). The ratio of demands to resources for the percentage contribution to R^2^ was therefore 6:1.

Table 3a shows the R^2^ values for the SEM inner model. Table 3b describes the path coefficients and impacts of the variables on negative acts.

The relative GoF is finally a means to validate a PLS-PM globally [35]. The relative GoF value, being 0.966 and thus bigger than 0.9, met the rule of thumb formulated by Vinzi et al. [36].

#### 3.3.2. Administrative versus Scientific Staff

Results from the SEM models for administrative and scientific personnel separately confirmed the general result that both resources and demands have significant effects on negative acts (H1) and that those effects are different between the two groups (H2a). The assessment of the GoF for both the measurement and reflective models and on the models for administrative and the scientific employees, respectively, was satisfied.

The structural model for the administrative staff showed that the latent variable of demands gave an effect on negative acts that was about 2.5 times stronger than the effect of resources, but even so, both effects were highly significant (Table 4). The gap in significance was instead accentuated when it comes to the scientific staff, and the effect of resources was not as significant as the effect of demands on negative acts, which was about five times bigger, or about twice that of the administrative personnel. This was also well expressed by the ratio of demands to resources for the contribution to R^2^, which was 3:1 for the administrative staff and about 7:1 for the scientific staff.

The effect of demands on bullying were therefore stronger for the scientific staff than for the administrative staff, while the effect of resources to prevent bullying was stronger in the administrative group than in the scientific group, thus confirming H2b and H2c.

When it comes to the measurement model, we also observed that demands were mainly constituted by institutional stress and lack of cooperation in terms of both weight and correlation. This was true for both the administrative and the scientific personnel, as well as for the general model. The patterns varied for the resources latent variable. In the model for the scientific staff, this was mainly formed by social support and secondly by autonomy, which was also observed for the model for the university employees as a whole. Instead, the results for the administrative staff indicated that meaningful job tasks were also weighted similarly to the other two observed variables (i.e., social support and autonomy), thus playing a relevant role in both the measurement and structural models. The added role of meaningful job tasks explained the stronger effect that resources had on negative acts for the administrative personnel.

## 4. Discussion

In this study, we aimed to determine the risk factors for being exposed to negative acts or bullying in different groups of personnel in the university sector. A main goal with examining specific risk factors for different positions is that it is possible to develop better initiatives aimed at preventing and reducing bullying for the different types of personnel and positions, thereby also offering employees in the university sector a healthier and safer work environment. There have been many suggestions for the potential risk factors for why the rates of self-reported bullying in universities are higher than in other work sectors, ranging from personal traits and high levels of competition among employees, lack of professional support, and poor leadership structures to neoliberalism taking over the university sector, thus depriving scientific personnel of important autonomy and influence and creating frustration and conflicts [37].

The results from the descriptive analyses for both scientific and administrative staff showed that these two groups of personnel experience significantly different levels of negative acts and self-reported bullying. The findings therefore support our hypothesis that there is a different risk of being exposed to bullying and negative acts at work depending on the type of profession, the job position, and the work task that employees are responsible for at the university. When considering the percentage of employees in both groups confirming being exposed to negative acts at the workplace or self-reported bullying in the last six months, we found that the scientific personnel had consistently higher values than the administrative personnel at 6.4% in the scientific group and 2.6% in the administrative group. The self-reported bullying rate was comparatively lower than the national average of 13%. A possible explanation for this is that the surveyed university has worked constructively over the past decade to counter workplace bullying and negative acts, thus reducing self-reported bullying from 11% at the highest measurement in 2009 down to 4.8% at the lowest in 2017.

Our results shows that the values were also significantly different between the two groups of personnel for 5 of the 12 negative acts questions. The scientific personnel reported significantly higher scores than administrative personnel on the following negative acts at work: hostility or silence in response to questions or attempts at conversation, having your opinions ignored, having gossip and rumours spread about you, having insulting or offensive remarks made about your person, attitudes, or your private life, and being ignored or excluded from the social community at work. All these negative acts are mainly person-related types of negative acts at work.

One interesting question one may ask here is why scientific personnel are more at risk of being exposed to bullying and negative acts in the workplace. The universities in Norway have a management culture where especially managers of scientific staff have a responsibility for many employees, and in our dataset, having between 50 to over 100 employees reporting to one leader is not uncommon, making it hard for these leaders to follow individual problems and conflicts on a day-to-day basis. Administrative leaders in universities, however, normally have considerably fewer employees per leader, and having over 20 employees is regarded as a big group for administrative leaders. In Norway, managers of scientific personnel are given a fixed-term position for 4 years, with the possibility to apply for two more periods. They must have a relevant PhD to obtain a leader position, but unfortunately, previous management experience is not always a job demand and can vary. Therefore, one may have leaders with a large span of employees and limited leadership experience. This in combination with more rotation in the leadership positions for scientific personnel than for administrative personnel might explain some of the differences found between the groups in our study.

In scientific positions, there are also some potential stressful demands such as uncertainty regarding receiving a permanent job position from Ph.D. positions to tenure tracks, high requirements regarding students’ feedback and evaluations, and measurements of research production. There is also a high degree of competition to obtain Ph.D. students and external funding, with much effort placed on applications despite the often-limited chance of their success. All of this may lead to a higher stress level in general among individual employees in scientific positions. Administrative staff can also have stressful work demands, but as a rule, there seems to be less competition among them for the same resources, and they are often in regular positions and experience more cooperation towards common goals, thus reducing the risk of being exposed to bullying and negative acts at work.

Universities have also been described as large and hierarchical organizations with an unequal distribution of power across positions [38], giving some members of certain professions the authority to behave in dysfunctional ways towards other employees without any negative sanctions or consequences, and there is some evidence that bullying is more frequent in these types of organizations [39].

One interesting finding in our results is the big difference in the models measuring how job demands and resources influence bullying among administrative personnel compared to scientific personnel. In both groups, job demands had a much stronger effect on bullying than job resources. Job demands in our study were represented by quantitative job demands, institutional stress, lack of cooperation, and lack of feedback. In our model, demands had a 2.5-fold stronger effect on the level of perceived negative acts than resources among administrative personnel.

When it comes to the results for the scientific personnel, the effect of resources was not as significant as the effect of demands on bullying, which was about five times greater. We argue that such a difference between the two groups of personnel can be explained by the differences in demands and resources in the job tasks and roles of the different positions, and thus, we claim that to reduce bullying, one must be aware of what types of demands and resources one needs to minimize or maximize for different types of employees. Our results also show that resources were not significantly related to bullying in the group of scientific personnel. This is a surprising finding considering the importance that resource factors including meaningful job tasks, autonomy, and social support have shown in theories on work motivation and well-being [40]. The results among the administrative personnel were different, and in this group, resources were significantly negatively related to bullying.

Another interesting finding in our study is that the variable of institutional stress seems to be an important job demand that predicts bullying among scientific personnel. Institutional stress is related to disagreeing with the organization’s policy, with the amount of power and influence one has as an employee, having conflicting values with those of the organization, and feeling that the organization uses the wrong parameters to measure the quality of one’s work [30]. A move towards a more business-like organization has challenged the everyday management of universities, and not all professionals agree on the decisions that are taken and do not perceive some of the outcomes as desirable.

Institutional stress may lead to a conflict between different worldviews in the academic sector, typically observed between those who manage, plan, and propose and those who are expected to follow and respond based on their views regarding different rationalities. For example, at a university, it is an important value and goal for most researchers that their research be free from leaders’ influence and be based on the interests, competence, profession, and knowledge of the researchers’ themselves. However, to finance one’s research, one is dependent on external funds, applications, and leadership steering the direction of research towards certain goals, values, and outcomes. In this landscape, organizational changes such as quality reforms in teaching and new rules and regulations regarding research time and funding can lead to a changing role of scientific positions, and scientific professionals can perceive demands coming from top management and political levels as a threat to the principle of academic freedom and independence in scientific positions. Some scholars have claimed that radical governmental changes have undermined important professions such as professors and doctors in public organizations [41]. The changing context in higher education in general with a growing knowledge of society, global flow, academic mobility, and international academics combined with the changing management style in universities in Scandinavia such as being more bureaucratically oriented and a growing level of political control and demands over important leadership factors at universities may in turn lead to lower levels of experienced academic autonomy. This may change the resource supply and increase levels of demands for different professions in the university sector and therefore the incidence of bullying.

## 5. Conclusions

In some organizations, bullying and other forms of harassment seem to be more or less “allowed” because such events are met with higher levels of tolerance than in other work environments. In universities, there can sometimes be a fine line between critical thinking that is important in academia and personal harassment.

Our findings indicate that structural organizational resources and demands predict a level of workplace bullying among university personnel and that scientific staff as a group experience different effects of demands and resources on bullying than administrative staff. There is also a significantly higher risk of experiencing both negative acts at work and self-reported bullying in a scientific position. Our findings also show that the effect of demands on bullying is stronger for the scientific staff than for the administrative staff.

Although management has little control over individual personality traits and characteristics, except in recruitment and promotion decisions, work environment factors are to a greater degree under the control of management, who may exert considerable influence on organizational structures, workflow, information flow, reward systems, job design, leadership training, conflict resolutions, and ensuring negative consequences for negative behaviours [42].

Future research based on our results should focus on how improved resources and increased demands for different professions in the university sector can decrease levels of bullying and negative acts. Our results indicate that an open, inclusive, and transparent management style when it comes to organizational changes and new requirements that affect employees’ everyday work can reduce institutional stress. A focus on how to create healthy, challenging, and supportive networks and groups where one can reduce individual competition and increase reward collaboration in research and teaching can be a good measure for both administrative and scientific staff.

A goal to reduce bullying and harassment must be central to all organizations because an average of 13% of employees report being bullied at work in general in the Norwegian university sector [14], and this is a serious concern for employees’ health. Previous research from Sweden has also indicated that work environment problems are strongly associated with production loss in academia [42] because it is difficult for employees to concentrate on performing important work tasks such as teaching and research if they are exposed to high levels of negative acts at work.

## Figures and Tables

**Table 1:** Principal component regression results of the pre-bullying (NAQ-R) variables that predict self-reported bullying.

NAQ-R Questions	Correlation	Value	Standard Error	t	Pr > |t|
(NAQ-1) Someone withholding information that affects your job performance (work-related bullying)	0.350	−0.023	0.033	−0.688	0.492
(NAQ-2) Repeated reminders of your errors or mistakes (person-related bullying)	0.435	0.045	0.037	1.219	0.223
(NAQ-3) Persistent criticism of your job efforts (work-related bullying)	0.430	0.081	0.036	2.248	0.025
(NAQ-4) Having been deprived of important responsibilities, being ordered to do work below your level of competence (work-related bullying)	0.376	0.075	0.032	2.335	0.020
(NAQ-5) Being shouted at or being the target of spontaneous anger (physical intimidating bullying)	0.421	0.086	0.033	2.642	0.008
(NAQ-6) Hostility or silence in response to questions or attempts at conversation (person-related bullying)	0.488	0.104	0.038	2.781	0.006
(NAQ-7) Having your opinions ignored (work-related bullying)	0.425	−0.013	0.037	−0.338	0.736
(NAQ-8) Having gossip and rumours spread about you (person-related bullying)	0.543	0.208	0.039	5.383	<0.0001
(NAQ-9) Having insulting or offensive remarks made about your person, attitudes, or private life (person-related bullying)	0.496	0.057	0.039	1.474	0.141
(NAQ-10) Being the subject of excessive teasing and sarcasm (person-related bullying)	0.446	0.174	0.036	4.856	<0.0001
(NAQ-11) Being ignored or excluded from the social community at work (person-related bullying)	0.472	0.157	0.034	4.644	<0.0001
(NAQ-12) Practical jokes carried out by people you do not get along with (person-related bullying)	0.350	−0.085	0.035	−2.458	0.014

**Table 2:** (**a**) Significant differences in response between the two groups of employees for both self-reported bullying and observed bullying from other colleagues. (**b**) In total, the scientific group had experienced five of the twelve types of negative acts at the workplace at a significantly higher rate than the administrative group. The scientific staff showed consistently higher values than the administrative staff.

(**a**)						
	**Self-Reported Bullying**			**Observed Bullying**		
Scientific Staff	6.4			23.8		
Administrative Staff	2.6			14.1		
Difference	3.8			9.7		
Significance	X			X		
(**b**)						
	**NAQ-1**	**NAQ-2**	**NAQ-3**	**NAQ-4**	**NAQ-5**	**NAQ-6**
Scientific Staff	42.7	14.2	14.2	16.7	18.0	19.8
Administrative Staff	37.0	14.1	10.9	16.4	16.7	12.5
Difference	5.7	0.1	3.3	0.3	1.3	7.2
Significance						X
	**NAQ-7**	**NAQ-8**	**NAQ-9**	**NAQ-10**	**NAQ-11**	**NAQ-12**
Scientific Staff	35.6	20.0	17.1	8.4	13.8	8.9
Administrative Staff	23.8	9.3	9.3	8.0	7.4	7.4
Difference	11.8	10.7	7.8	0.4	6.4	1.5
Significance	X	X	X		X	

**Table 3:** (**a**). Significance and R^2^–values for the SEM inner model. (**b**) Path coefficients and impacts of the variables on negative acts at workplace.

**(a)**					
**R^2^**	**F**	**Pr > F**	**R^2^ (Bootstrap)**		**Standard Error**
0.447	371.893	0	0.455		0.03
**(b)**					
**Latent Variable**	**Value**	**Standard Error**	**t**	**Pr > |t|**	**Contribution to R^2^ (%)**
Resources	−0.146	0.031	−4.664	0	14.331
Demands	0.568	0.031	18.136	0	85.669

**Table 4:** SEM results for the scientific and the administrative personnel.

	Latent Variable	Value	Standard Error	Pr > |t|	Correlation	Path Coefficient	Contribution to R^2^ (%)
**Administrative personnel**	Resources	−0.193	0.057	0.001	−0.497	−0.193	24.686
	Demands	0.483	0.057	0.000	0.604	0.483	75.314
**Scientific personnel**	Resources	−0.121	0.045	0.007	−0.487	−0.121	13.016
	Demands	0.591	0.045	0.000	0.666	0.591	86.984

## Data Availability

The data presented in this study are available on request from the corresponding author. The data are not publicly available due to use of external partner for data collection, data anonymization and data storage.
